# Effects of coarse cereals on dough and Chinese steamed bread – a review

**DOI:** 10.3389/fnut.2023.1186860

**Published:** 2023-08-03

**Authors:** Yunfei Yang, Xinwei Wang

**Affiliations:** College of Food Science and Technology, Henan University of Technology, Zhengzhou, China

**Keywords:** coarse cereals, nutrition, dough, Chinese steamed bread, quality analysis

## Abstract

Chinese steamed breads (CSBs) are long-established staple foods in China. To enhance the nutritional value, coarse cereals such as oats, buckwheat, and quinoa have been added to the formulation for making CSBs. This review presents the nutritional value of various coarse cereals and analyses the interactions between the functional components of coarse cereals in the dough. The addition of coarse cereals leads to changes in the rheological, fermentation, and pasting aging properties of the dough, which further deteriorates the appearance and texture of CSBs. This review can provide some suggestions and guidelines for the production of staple and nutritious staple foods.

## 1. Introduction

Chinese steamed breads (CSBs) are traditional staple food in north China, also called “mantou.” CSBs are great energy supplement because of their simple recipe and easy availability of ingredients. The food industry has evolved over thousands of years, CSBs are still one of the most critical foods in China (1). Recently, CSBs have attracted the extensive attention of researchers. According to existing papers, the raw materials of traditional CSBs are mainly wheat flour (WF), water, and yeast or sourdough (2). Generally, the production process involves mixing the ingredients, kneading the dough, proofing it, and then steaming it (3).

As consumers’ dietary concepts gradually move toward low sugar and rich nutrition, traditional CSBs are increasingly considered not a portion of healthy food. It has a high glycemic index, which is not friendly to diabetics and consumers who need to lose weight (4). Considerable researches have emerged on how to improve the nutritional quality of the CSBs while maintaining their appearance (5). The most widely studies are adding coarse cereals flour to wheat flour so as to achieve the purpose of improving the nutritional properties of CSBs. While changing the nutrition of CSBs, phenomena in the production process deserve to be systematically analyzed. The interaction of different components with wheat flour affects the dough characteristics, and the change in dough characteristics also impacts the properties of the CSBs.

Coarse cereals have a variety of definitions in relevant studies. Some researchers believe that grain crops other than rice and wheat are counted as coarse cereals, such as corn, oats, sorghum, etc. (6). Some researchers think that coarse cereals refer to crops other than the five major crops of rice, wheat, corn, soybean, and potato, mainly include sorghum, millet, buckwheat, oats, etc. (7). This paper uses the former definition for the statistical analysis of these coarse cereals.

In this review, the nutritional components of coarse cereals were statistically analyzed, followed by the effect of the addition of various coarse cereals to wheat flour on the rheological, fermentation, and pasting characteristics of the dough, as well as the appearance and textural characteristics of the corresponding coarse cereals CSBs. Following these conclusions, the article discusses the trends and directions of the relevant industries.

## 2. Nutritional properties of coarse cereals

As shown in the Table 1 shown, compared to wheat, coarse cereals usually contain different types of proteins with good amino acid composition (10, 26). The fiber, unsaturated fatty acids, and bioactive substances in coarse cereals are also beneficial to the body (27–30), and from this perspective, the addition of coarse cereals to pasta products is similar to the development of whole-wheat flour foods, both of which are designed to provide consumers with nutrients that are less abundant in refined flours, such as dietary fiber, resistant starch, and polyphenols (31, 32).

**TABLE 1:** The unique nutritional value of various coarse cereals.

Name of cereals	Nutritional ingredient	References
Corn	Dietary fiber, vitamin B, vitamin E, carotene, luteinizing hormone, phosphorus, magnesium, potassium, zinc	(8, 9)
Oat	Essential amino acids, fatty acids, soluble dietary fiber, mixed beta-glucans	(10, 11)
Buckwheat	Resistant starch, dietary fiber, low soluble-insoluble dietary fiber ratio, amino acid balance	(12, 13)
Sorghum	Phenolic compounds, sorghum protein, slow-digesting starch	(14, 15)
Millet	Phytochemicals, phenolic, phytic acid, dietary fiber, iron, calcium, vitamin B	(16, 17)
Quinoa	Unsaturated fatty acids, vitamins (folic acid and tocopherol), minerals, dietary fiber, polyphenols	(18, 19)
Barley	Soluble fiber, β-glucan, phenolic compounds.	(20, 21)
Potato	Balanced amino acid composition, total vitamins, minerals, flavonoids, polyamines, carotenoids	(22, 23)
Sweet potato	Dietary fiber, minerals, vitamins, polyphenols (anthocyanins and other polyphenols)	(24, 25)

Besides, many kinds of cereal contain higher levels of phytochemicals than wheat, especially polyphenols (14). Polyphenols can scavenge free radicals in the body to achieve the effect of antioxidants (33), polyphenols can also play a role in lowering blood pressure, blood lipids, and cholesterol, it has a preventive effect on cardiovascular and cerebrovascular diseases and atherosclerosis (34–36). These polyphenols may also promote the slow digestion of starch by inhibiting digestive enzymes (37). Polyphenols also contribute to health protection and antioxidant performance. These dietary antioxidants can prevent oxidative stress, oxidative damage caused by poor diet, and exposure to ultraviolet rays (38).

In addition, most coarse cereals contain higher dietary fiber content (39), which could promote the gastrointestinal tract peristalsis, accelerate the digestion and absorption of food, help the excretion of feces, and has a therapeutic effect on constipation (40). After entering the body, dietary fiber can also stabilize intestinal flora, thus regulating immunity (41). Dietary fiber can also reduce the rate of sugar intake, slow the rise of blood sugar, and control blood sugar concentration, suitable for diabetic patients (42), also dietary fiber has a strong sense of satiety, making it an ideal food for weight loss periods (43). Adding coarse cereals to flour reduces starch digestibility, increases vitamin and mineral content, lowers food GI, and improves nutritional function, all of which will bring more healthy food to consumers (44).

## 3. Interaction of specific components of coarse cereals in the dough

When coarse cereals and wheat flour are used to make CSBs, not only the nutritional value of steamed bread changed, but also many changes can be observed in the process of making CSBs. These interrelated changes are reflected in dough morphology, rheological indexes, and steamed bread properties (45–48).

In the traditional wheat flour dough, gliadin is a monomer protein with a small molecular weight (49). The components are connected by hydrogen bonds, and intramolecular disulfide bonds to form a compact three-dimensional structure, which provides adhesion and ductility for the dough (50). Gluten proteins contain glutenin and gliadin. During dough formation, disulfide bonds between protein peptide chains and within the molecule are held together by external forces to form macromolecules, creating a dough with ductility and elasticity (51). Gluten protein makes the dough water holding, adhesive and viscoelastic, which plays a decisive role in the quality of flour products. However, when coarse cereals flour is added to wheat flour to form a dough, coarse cereals will hinder the formation of gluten network structure and reduce the strength of gluten network structure (52–55).

Dough rheological properties and the properties of CSBs were affected when adding coarse cereals (28, 53, 56). The specific influence mechanism is mainly divided into the following aspects:

### 3.1. The interaction between phenols with other components in dough

Oats, purple potatoes, mung beans, and other crops contain a large number of polyphenols, and the molecular side chain of phenols has a large number of hydroxyl groups. Hydroxyl groups belong to hydrophilic groups, which will compete for water with substances such as starch and protein. Polyphenols rich in flour will increase the water absorption of dough, resulting in a series of changes in the process of making CSBs. It has been reported that the hydroxyl and carboxyl groups of dissolved phenolic compounds could interact with water and indirectly interact with the hydroxyl groups of starch through a hydrogen bond, to change the properties of water and starch (37). Water soluble phenolic compounds will significantly affect the gelatinization temperature and peak viscosity of starch. But some water-insoluble phenolic compounds, such as rutin, have no significant effect on the gelatinization performance of starch (57). Therefore, the solubility of phenolic compounds may be one of the factors affecting the gelatinization of starch when adding coarse cereals (58).

In addition to the binding effect with water, it has been shown that phenolic compounds could hinder the formation of disulfide bonds between proteins, which is detrimental to the formation of protein network structures, leading to the reduction of dough strength, gas holding capacity, and specific volume of products (59). In addition to affecting the rheological properties of dough, the nutritional value of phenols in food attracts the interest of researchers, plant phenolic compounds can inhibit α-glucosidase activity, slowing down starch decomposition, and significantly reducing starch hydrolysis rate (60). But some studies have found the interaction of proteins with phenols leads to changes in the total phenolic or flavonoid content, and a reduction in antioxidant activity, contrary to what the addition of cereals is intended to achieve (24, 60).

### 3.2. The interaction of enzymes with other components in dough

Enzymes often play a vital role in the food system. Normally we separate the enzymes in food into endogenous enzymes and added exogenous enzymes, both of them have an important effect on the quality of cereal foods (32, 61). Lipase, amylase, and xylanase are common enzymes in cereals. The amylase enzyme is rich in oats, buckwheat, quinoa, etc. (59, 62). For example, when flour is rich in α-amylase, the gelatinization and retrogradation characteristics of dough are considerably changed (44). Some researchers have found that the addition of amylase and enzyme combinations of α-amylase (6 and 10 ppm), xylanase (70 and 120 ppm), and cellulase (35 and 60 ppm) can improve the extensibility, softening, mixing tolerance index (MTI) and stickiness of bread dough (63).

In addition to this, the texture of the CSBs will be affected accordingly. During CSBs storage, the dextrinized starch (branched starch) network present in fresh CSBs gradually transforms into an extensive, partially crystallized branched starch network with original branched starch microcrystals acting as linkage zones (19, 64). In addition, as starch aging continues, water migration occurs in the starch grain structure and more and more water is immobilized in the branched starch microcrystals (65, 66). The overall class mobility of the CSBs decreases due to the reduced flexibility of the gluten network, which is closely related to the increased crumb stiffness and reduced crumb elasticity (67).

### 3.3. The interaction of non-starch polysaccharides with other components in dough

In the food system, non-starch polysaccharide (NSP) is mainly composed of cellulose, hemicellulose, pectin, and resistant starch. It can be divided into insoluble NSP and soluble NSP. In the process of making steamed bread, the non-starch polysaccharides that play a role mainly include dietary fiber, β-glucan (BG). Fiber is divided into soluble dietary fiber and insoluble dietary fiber. A large number of studies show that the addition of dietary fiber will increase the water absorption of dough (20).

Soluble dietary fiber will intercept digestive enzymes after water absorption and expansion, thus inhibiting carbohydrate digestion and reducing GI. Hydrocolloids can strongly affect the secondary conformation of proteins and the hydration properties of gluten. As a kind of hydrocolloid, BG is tightly bound with large amounts of water in the dough. BG competes with protein for water absorption, which is detrimental to the formation of the gluten network and affects the dough formation time and stability.

## 4. Effect of coarse cereals addition on characteristics of dough and CSBs

**FIGURE 1:**
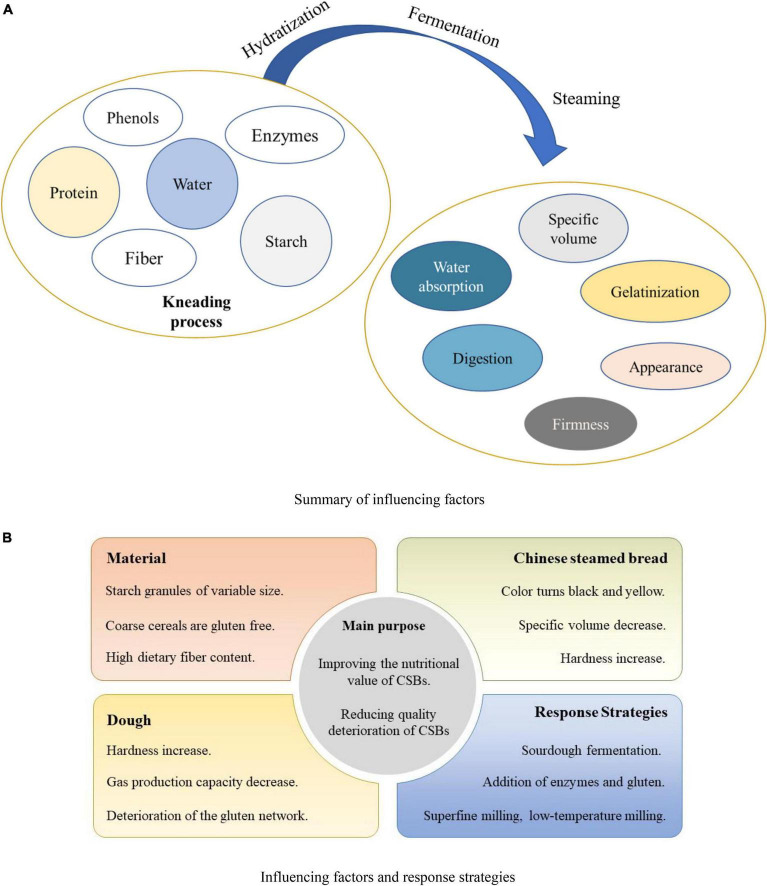
Key factors and procedures in the Chinese steamed breads (CSBs) making process. **(A)** Summary of influencing factors. **(B)** Influencing factors and response strategies.

As mentioned above, the process of producing traditional steamed bread is mixing, kneading into a dough, proofing, and steaming (3). With the addition of coarse cereals, the properties of the dough and CSBs are affected accordingly due to the change of starch, protein and other components, and the key factors are shown in Figure 1. In order to improve the product quality to some extent, many researchers have also optimized the production process, such as changing the traditional primary fermentation to secondary fermentation and using the sour dough fermentation method to make CSBs (2).

### 4.1. Effect of coarse cereals addition on characteristics of dough

Dough in pasta processing is a complex mixture of flour, water, yeast, salt, or other ingredients and is the essential transition form of wheat from flour to food (68). When wheat flour was watered to about 50% and kneaded, cohesive and elastic dough was obtained (69). The structure and properties of the dough, especially the rheological properties, are vital for the production of pasta for two main reasons: firstly, the property of the dough depends on the quality of the wheat flour. The structure of the starch and protein affects the flour properties, tensile properties, kneading and mixing properties, and the fermentation properties of the dough (70, 71). Secondly, the properties of the dough directly affect the quality of products such as steamed bread and noodles. The rheological properties of the dough determine the quality of the dough during mechanical processes such as dough proofing, dividing and rounding (72). By improving the recipe and adjusting the processing, the characteristics of the dough can be controlled to produce CSBs that meet specific quality requirements (73).

Therefore, the research on dough products cannot avoid relating all of these indicators. Most of the researchers use rheological indicators such as dough flouriness, stretching, kneading, and foaming as the leading quality indicators.

#### 4.1.1. Effect of coarse cereals on rheological properties

Rheology is a subject that studies the flow and deformation of materials. In the load curve of dough, the relationship between stress, strain, and time and the resulting elasticity, viscosity, plasticity, and other properties are called the rheological properties of dough (69, 74).

In the process of kneading wheat flour into dough with water, the instruments used to reflect and test the rheological properties of dough mainly include the farinograph (75). The indicators tested by the farinograph are as follows: water absorption, dough development time, stability time, and breakdown time (54, 76).

Dough rheological properties are affected when coarse cereals flour is added, this may be caused by the weakening of protein cross-linking due to the high fiber content in coarse cereals competing for water. Similar effects have also appeared in other studies. Compared with wheat, millet have higher water absorption, water solubility, and oil absorption index (45). The water absorption of the dough is directly proportional to the dough development time and dough stability time. When the water absorption decreases, the dough development time is usually shortened due to the disruption of the continuous gluten network structure, which means that the dough strength decreases. Millet interacts strongly with water and oil, and considering this factor alone, adding millet flour seems to make the dough and CSBs properties better, but adding millet flour to the dough will dilute the concentration of gluten protein, which will lead to a decrease in the strength of the gluten structure in the dough and thus deteriorate the quality of the CSBs (77).

Researchers conducted numerous investigations into the mechanisms behind the change in dough properties. With the addition of oat flour and oat bran, the β-glucan content of the mixed flour dough increased significantly. As we have mentioned that β-glucan could interact with water and result in higher water absorption. As with many other indicators, water absorption is not influenced by one cause only (78, 79). Once coarse cereals flour was added to wheat flour, the water absorption of the dough increased and the gluten content decreased, and these changes occurred simultaneously. In general, the deterioration in quality due to the decrease in gluten content was more obvious.

This phenomenon can be corroborated by the breakdown values, as more flour was replaced, the content of gluten protein in the mixed flour became less, which gradually reduces the viscoelasticity of the dough. Therefore, in the viscosity measurement, the viscosity of the mixing system of flour and water became weaker and more prone to collapse. Thus, the breakdown value becomes larger.

As Table 2 shows, the addition of millet flour to wheat flour will reduce the dough development time which is similar to other cereals, which is due to the decrease of gluten content. After the addition of millet flour, it is observed that the dough stability and dough development time are positively correlated with the mixed wet gluten index (45, 80). When quinoa flour was added, the breakdown value decreased with the increasing amount of quinoa flour added, it represents that after the addition of buckwheat flour, the dough’s ability to resist shear and withstand heat was significantly higher than ordinary dough (46, 81).

**TABLE 2:** The influence of adding coarse cereals on the dough and Chinese steamed bread (CSB).

Name of cereals	Main ingredients	Addition levels	Influence on the dough	Influence on the CSB	References
Corn	Purple corn flour (different particle sizes)	100% PCF to make dough and CSB.	Hardness increases (wheat dough: 0.2 N; PCF dough: 48.30 N).	Specific volume decreases (wheat CSB:3.27 mL/g; PCF CSB:1.20 ∼ 1.34 mL/g); Hardness increases (wheat CSB:40.53 N PCF CSB: 32.97 ∼ 67.76 N).	(88)
	High-amylose maize starch (HAMS)	HAMS/WF at weight ratios of 0/100, 2/98, 4/96, 6/94, 8/92, and 10/90.	Water absorption increases. Decrease in extensibility.	Specific volume decreases (from 2.01 mL/g to 1.59 mL/g), and rate of staling decreases (from 1.389 to 0.729); hardness increases (from 539 to 805 g).	(89)
Oat	β-glucan from oat	0.0–5.0% (wheat flour basis).	Water absorption increases (from 57.0 to 69.0%). Peak viscosity decreases (from 487.9 to 329.0 cp).	Specific volume increases then decreases (0%:2.43 mL/g; 2%: 2.64 mL/g) Hardness increases (0%:1078 g; 2%: 1562 g)	(90)
	Ground oatmeal	Wheat flour was replaced with 0, 10, or 30% of ground oatmeal	/	Hardness increases (0%:1188.98 g; 30%:1705.08 g). Aging rate reduces.	(53, 79)
Buckwheat	Tartary buckwheat bran flour (TBBF)	TBBF-WF flour blend (W_*bran*_ = 0, 10, 30, 50, 70, and 90%)	Water absorption decreases (from 59.3 to 55.9%). Development time increases (from 3.29 to 0.97 min)	L* value decreases (from 81.88 to 38.49). Hardness increases (from 1651 to 19691 g).	(79)
	Buckwheat flour	0, 5, 10, 15% w/w based on wheat flour dry weight	Water absorption increases (from 63.7 to 71.4%). Stickiness decreases (from 46.22 to 27.59 g).	Specific volume decreases (from 2.47 to 1.69 mL/g). Hardness increases (from 228 to 687 g).	(13)
Sorghum	Whole grain red sorghum (WwhSorg), Commercial red sorghum (ComRSorg)	Sorghum flour: sourdough = 17:6	/	L* value decreases (Commercial wheat:77.83; WwhSorg:64.47;ComRSorg:55.57)	(46)
Millet	Millet flour (MF)	25% millet flour in substitution of wheat flour	Water absorption decreases (from 56.8 to 52.0%). The FQN decreases (from 107 to 85)	Hardness increases (from 1786 to 2764 g).	(45)
	Dietary fiber (DF) from millet bran	0, 2%, 4%, 6%, 8%, and 10% (w/w)	Water absorption decreases (from 56.8 to 51.9%). The FQN decreases (from 107 to 83)	Hardness increases (from 1786 to 5351 g). The L* value decreases (from 92.32 to 91.76).	
Quinoa	Quinoa flour (QF)	QF/WF: weight ratios of 85/15, 70/30, 55/45, 40/60, 25/75 and 10/90.	L* value decreases (from 81.24 to 75.6), ΔH increases (from 5.5 to 7.5)	L* value decreases (from 85.58 to 68.19) After adding more than 15% QF, the hardness and chewiness increased.	(91)

The main effect should be attributed to starch, crops contain different starch, mainly reflected in starch particle size, starch type (amylose and amylopectin), and damaged starch content. As there are significant differences in starch particle size, crystallinity, fine structure, and straight chain starch content between sources, which can cause large differences in pasting parameters (64, 80, 82, 83). The thermodynamic properties of different types of starch are completely different, which can affect the viscosity and gluten strength of the dough during the processing stage, thereby affecting the final product quality; The appropriate size of starch particles can fill the pores in the three-dimensional network structure of the protein, thus enhancing the strength of gluten (17, 84). All these reasons lead to the changes in dough fermentation and rheological properties, which affect the quality of steamed bread. For the development of the coarse cereals flour products industry, it is essential for researchers to analyze and explore countermeasures.

Starch from various sources has different qualities, especially in gelatinization characteristics, crystal structure, particle size, chain length distribution, and anti-enzymatic hydrolysis characteristics. If there is a discrepancy between the indicators mentioned above, in the human body, the rate of digestion of starch changes as well (85, 86).

When analyzing the rheological properties of doughs, the indicators water absorption (WA), dough development time (DDT), and farinograph quality number (FQN) are interlinked. When researching on coarse cereals pasta products, it is essential not only to analyze the effects of the components of the cereal but also to link these indicators together and analyze the specific patterns that can be applied in future studies.

#### 4.1.2. Effect of coarse cereals on gelatinization and retrogradation characteristics

The gelatinization characteristics of wheat flour were measured by running a heating-holding-cooling program with a viscometer and recording the torque magnitude. In this testing process, the pasting temperature, peak viscosity, peak viscosity time, breakdown value, holding viscosity, final viscosity, and setback value of flour or starch can be obtained through data processing (87).

As shown in Table 2, the addition of coarse cereals flour can significantly reduce the gelatinization enthalpy of the dough, which may be caused by irregular starch granules.

The analysis of a large number of articles showed that the type (corn, wheat, etc.) and characteristics (particle size, damaged starch content, etc.) of the starch have a significant influence on these indicators. when the starch particles are uniform, it seems that researchers easier to get a regular and characteristic result, when the size and shape of starch particles are different, their gelatinization and retrogradation characteristics will be more difficult to predict (38).

#### 4.1.3. Effect of coarse cereals on fermentation characteristics

In addition to the kneading properties and stretching properties of the dough, the fermentation properties are also important when making bread and other fermented products. The dough produces CO_2_ gas during the fermentation process to obtain a loose and porous dough structure, and a good fermentation can significantly improve the quality of the bread (92). A high quality fermentation means that the proteins and starches should fully absorb water and cross-link with each other, which is macroscopically manifested by a dough that can be pulled into a thin film. In the experiment, Brabender’s rheological fermentograph was used to test the fermentation performance of the dough.

In general, the addition of coarse cereals had a negative effect on most of the characteristics of the dough. However, during the analysis, certain indicators showed a trend in favor of the dough. For example, the water absorption of the dough increased with the addition of most of the coarse cereals (88). Water holding capacity is usually used as a measure of protein and fiber absorption and water retention. High water holding capacity means high moisture content in the dough, which improves the taste and texture of the flour product (79).

However, all the previously mentioned factors should be considered as a whole, and observing and using these patterns to optimize the recipe is the purpose and focus of this study.

### 4.2. Effect of coarse cereals on the quality of CSBs

When making different types of CSBs, the quality of the final product is generally evaluated by texture indicators such as color, specific volume, aspect ratio, hardness, elasticity, resilience, and chewiness of the CSBs. The color of CSBs is the most intuitive indicator. Once the color of CSBs appears yellow or black, consumers will reduce their appetite and subjectively consider the quality of CSBs to be inferior.

When coarse cereals were added to enhance the nutritional value of the CSBs, the color of the CSBs will inevitably change, so how to mitigate the color deterioration is a priority when conducting related research.

In the texture properties of steamed bread, hardness, stickiness, and chewiness are negatively correlated with the quality of the steamed bread. When the values of these three indicators are large, the steamed bread will be hard, difficult to chew, and sticky when eaten. The quality of the steamed bread obtained, recovery, cohesion, and elasticity are positively correlated with the quality of the steamed bread.

#### 4.2.1. Effect of coarse cereals on CSBs appearance

When measuring the quality of CSBs, the first thing people notice is the color of the Chinese steamed bread, and the second is whether the steamed bread is full. In actual experiments, the degree of fullness is not easy to measure, so researchers often use specific volume and height-diameter ratios to describe whether the steamed bread is full or not.

A colorimeter has usually been used when measuring the color of CSBs, and researchers can get three indicators through the instrument: lightness (L*), redness-greenness (a*), and yellowness-blueness (b*). For the darker crumbs, some researchers believe that this is since that they have more prominent particles, which produce larger and darker shadows in the structure of the CSBs. In a related study of buckwheat CSBs, when the substitution level reached 70%, the brightness was significantly reduced by 28%.

In terms of color, after adding coarse cereals flour, especially Tartary buckwheat flour, the b* was markedly higher, and the L* and a* were significantly lower than those of ordinary wheat flour products, which indicated that the addition of Tartary buckwheat would make the product darker and light green.

Another reason for the reduction in brightness may be that the added oat flour is rich in BG. As shown in Table 2, when buckwheat flour rich in beta-glucan was added to the flour to make steamed bread, the brightness of the steamed bread core was improved.

#### 4.2.2. Effect of coarse cereals on CSBs textural characteristics

Texture analyzer is a kind of test for hardness, brittleness, elasticity, resilience, adhesiveness, cohesion, viscosity, bending ability, breaking/breaking force, brittleness, chewability, stickiness, tensile strength, elasticity, etc., these data were often used in experimental design to measure the quality of steamed bread.

Coarse cereals flour tends to have higher dietary fiber content, and as the level of addition increases, the textural properties of the steamed bread changed, Abundant studies have shown that the addition of coarse cereals flour changed the secondary structure of proteins and disrupts the network structure of gluten (28). This will result in increased hardness and decreased specific volume of pasta products (including but not limited to CSBs).

As mentioned earlier, the starch type is also an important factor, as shown in Table 2, with the addition of oat flour and the increase in dietary fiber content, the gluten network structure of the dough will be diluted, so the specific volume of steamed bread will drop significantly. When millet flour was added to the flour, the high content of branched chain starch and a large amount of starch dextrin dissociation when cooking was carried out reduces the strength of the protein network structure and made the CSBs less sticky. The addition of branched chain starch weakened the strength of the composite network of starch and protein and increased the air-holding capacity of the dough, and these changes led to a decrease in the hardness, viscosity and chewiness of the CSBs.

The addition of coarse cereals always leads to deterioration in the quality of CSBs, and this deterioration is usually related to the level of added cereals, some of which become unpopular with consumers because of their own anthocyanins and other substances, and some because the addition of coarse cereals dilute the gluten structure leading to a decrease in gas-holding properties, a decrease in CSBs specific volume and a darkening of color.

#### 4.2.3. Effect of coarse cereals on CSBs digestion characteristics

The purpose of many researchers utilizing mixed grains in the production of pasta products is to lower the GI value of the food and create foods suitable for special populations such as diabetics (4). *In vitro* digestion is usually used to simulate the process of food digestion in the human body (93), and the addition of coarse cereals results in an effective reduction of the eGI value of the food.

When adding potato flour to make CSBs, the particle size of potato starch is larger than wheat starch. As particle size increases, the surface area exposed to digestive enzymes decreases, resulting in lower digestibility. The eGI value of CSBs with 35% potato flour added can be reduced to 65.35 (94).

In addition, polyphenols represented by rutin were found to interact with α-amylase, α-glucosidase, and starch to form complexes that reduced starch digestibility, leading to a lower eGI value of the product (46).

## 5. Conclusion and prospects

Adding coarse cereals to staple foods is a reliable way to improve the nutritional content of staple foods. However, the inclusion of coarse cereals adversely affects the dough quality, particularly in relation to CSBs, as evidenced by research highlighting the deterioration of its visual and structural attributes, including darker color, diminished specific volume, and heightened hardness.

Conducting a thorough investigation into the magnitude of the deterioration and its underlying causes, as well as devising strategies to mitigate or eradicate the deterioration, is of utmost importance. Previous studies have explored potential solutions, such as incorporating emulsifiers and enzymes that are either absent or present in limited quantities in coarse cereals and flours.

In some recent studies, it has been found that the addition of xylanase improves product quality (63). Xylanase breaks down the cell walls of raw materials and β-glucan in the brewing or feed industry (95), reduces the viscosity of the material, promotes the release of active substances, and reduces non-starch polysaccharides in food, promote the absorption and utilization of nutrients such as soluble lipid components (96, 97).

Some other enzymes, such as lipase, can also improve the quality of the CSBs (49). Numerous studies have shown that adding lipase in appropriate amounts can improve the gluten strength, prolong the stabilization time, reduce the degree of weakening, increase the dough elongation and tensile resistance, and improve the rheological properties of the dough (98). The specific volume, color, structure, and flexibility of CSBs are all improved to a certain extent. And the amount of lipase added is not the larger the better, too much added will have a negative impact on the CSBs (74).

Not only adding enzymes can improve the quality of coarse cereals CSBs, but also the treatment of raw materials, such as ultra-micro-grinding and low-temperature milling of coarse cereals, it has been reported that the special milling method can effectively retain the unique flavor components in the plant, and the total flavonoids and polyphenol substances after grinding to get a more comprehensive release (99, 100). Besides, superfine milling treatment can make part of the fiber from insoluble to soluble, thus increasing the proportion of soluble fiber (101, 102), and as we have already concluded, appropriately increasing the content of soluble fibers will improve the quality of CSBs.

As mentioned above, fermentation is also an important part of the CSB production process and many studies on sourdough have been published. During the prolonged fermentation of sourdough, microbial metabolism and endogenous enzyme biotransformation provide sufficient time in which the metabolism of lactic acid bacteria produces organic acids, and microbial metabolism, microbial, andendogenous enzyme biotransformation contribute to changes in arabinoxylan, proteins, and starch, which improves the volume and texture of bread (2, 103). The main reason for the deterioration of coarse cereals CSBs is due to the complex composition of the dough which leads to a decrease in gas production during fermentation and a reduction in dough strength, which can be effectively reduced by sourdough fermentation.

In addition to studying the mechanism of quality change in coarse cereals CSBs, Also worthy of further study are the benefits of coarse cereals CSBs on the human body. Specifically, factors affecting the expect glycemic index (eGI) of coarse cereals CSBs, and the effects of long-term consumption of coarse cereals CSBs on diabetes, hyperglycemia, hypertension, and other diseases. It is believed that these studies can promote coarse cereals CSBs as a staple food for sub-healthy people.

In conclusion, coarse cereals CSBs are a promising healthy staple food due to the unique nutritional value of each coarse cereals. With specific descriptions of human health benefits and superior product quality, it is believed that coarse cereals CSBs will occupy a unique position in the market.

## Author contributions

YY: acquisition of data and drafting the manuscript. XW: revising the manuscript critically for important intellectual content. Both authors contributed to the article and approved the submitted version.

## References

[1] Zhu F. Staling of Chinese steamed bread: quantification and control. *Trends Food Sci Tech.* (2016) 55:118–27. 10.1016/j.tifs.2016.07.009

[2] Wang X Zhao R Yuan W. Type I sourdough steamed bread made by retarded sponge-dough method. *Food Chem.* (2020) 311:126029. 10.1016/j.foodchem.2019.126029 31874427

[3] Wu C Liu R Huang W Rayas-Duarte P Wang F Yao Y. Effect of sourdough fermentation on the quality of Chinese Northern-style steamed breads. *J Cereal Sci.* (2012) 56:127–33. 10.1016/j.jcs.2012.03.007

[4] Zhu F. Glycemic control in Chinese steamed bread: strategies and opportunities. *Trends Food Sci Tech.* (2019) 86:252–9. 10.1016/j.tifs.2019.02.038

[5] Lau E Soong YY Zhou WB Henry J. Can bread processing conditions alter glycaemic response? *Food Chem.* (2015) 173:250–6. 10.1016/j.foodchem.2014.10.040 25466020

[6] Zhang Y Capanoglu E Jiao L Yin L Liu X Wang R Coarse cereals modulating chronic low-grade inflammation: review. *Crit Rev Food Sci.* (2022) 174:114401. 10.1080/10408398.2022.2070596 35503432

[7] Kaur KD Jha A Sabikhi L Singh AK. Significance of coarse cereals in health and nutrition: a review. *J Food Sci Tech.* (2014) 51:1429–41. 10.1007/s13197-011-0612-9 25114333 PMC4108649

[8] Li X Wang C Krishnan PG. Effects of corn distillers dried grains on dough properties and quality of Chinese steamed bread. *Food Sci Nutr.* (2020) 8:3999–4008. 10.1002/fsn3.1604 32884681 PMC7455979

[9] Dahal A Sadiq MB Anal AK. Improvement of quality of corn and proso millet-based gluten-free noodles with the application of hydrocolloids. *J Food Process Pres.* (2021) 45:e15165. 10.1111/jfpp.15165

[10] Krochmal-Marczak B Tobiasz-Salach R Kaszuba J. The effect of adding oat flour on the nutritional and sensory quality of wheat bread. *Br Food J.* (2020) 122:2329–39. 10.1108/BFJ-07-2019-0493

[11] Ronda F Perez-Quirce S Lazaridou A Biliaderis CG. Effect of barley and oat β-glucan concentrates on gluten-free rice-based doughs and bread characteristics. *Food Hydrocolloid.* (2015) 48:197–207. 10.1016/j.foodhyd.2015.02.031

[12] Coțovanu I Mironeasa S. Buckwheat seeds: impact of milling fractions and addition level on wheat bread dough rheology. *Appl Sci.* (2021) 11:1731. 10.3390/app11041731

[13] Liu W Brennan M Serventi L Brennan C. Buckwheat flour inclusion in Chinese steamed bread: potential reduction in glycemic response and effects on dough quality. *Eur Food Res Technol.* (2017) 243:727–34. 10.1007/s00217-016-2786-x

[14] Yousif A Nhepera D Johnson S. Influence of sorghum flour addition on flat bread *in vitro* starch digestibility, antioxidant capacity and consumer acceptability. *Food Chem.* (2012) 134:880–7. 10.1016/j.foodchem.2012.02.199 23107703

[15] Kumar T Dweikat I Sato S Ge Z Nersesian N Chen H Modulation of kernel storage proteins in grain sorghum (Sorghum bicolor (L.) Moench). *Plant Biotechnol J.* (2012) 10:533–44. 10.1111/j.1467-7652.2012.00685.x 22353344

[16] Makokha AO Oniang’O RK Njoroge SM Kamar OK. Effect of traditional fermentation and malting on phytic acid and mineral availability from sorghum (Sorghum bicolor) and finger millet (Eleusine coracana) grain varieties grown in Kenya. *Food Nutr Bull.* (2002) 23(3 Suppl.):241–5. 12362804

[17] Li S Zhao W Li P Min G Zhang A Zhang J Effects of different cultivars and particle sizes of non-degermed millet flour fractions on the physical and texture properties of Chinese steamed bread. *Cereal Chem.* (2020) 97:661–9. 10.1002/cche.10282

[18] Xu X Luo Z Yang Q Xiao Z Lu X. Effect of quinoa flour on baking performance, antioxidant properties and digestibility of wheat bread. *Food Chem.* (2019) 294:87–95. 10.1016/j.foodchem.2019.05.037 31126509

[19] Wang XX Lao X Bao YZ Guan X Li C. Effect of whole quinoa flour substitution on the texture and in vitro starch digestibility of wheat bread. *Food Hydrocolloid.* (2021) 119:106840. 10.1016/j.foodhyd.2021.106840

[20] Demir G Klein HO Mandel-Molinas N Tuzuner N. Beta glucan induces proliferation and activation of monocytes in peripheral blood of patients with advanced breast cancer. *Int Immunopharmacol.* (2007) 7:113–6. 10.1016/j.intimp.2006.08.011 17161824

[21] Koletta P Irakli M Papageorgiou M Skendi A. Physicochemical and technological properties of highly enriched wheat breads with wholegrain non wheat flours. *J Cereal Sci.* (2014) 60:561–8. 10.1016/j.jcs.2014.08.003

[22] Liu XL Mu TH Sun HN Zhang M Chen JW Fauconnier ML. Comparative study of the nutritional quality of potato-wheat steamed and baked breads made with four potato flour cultivars. *Int J Food Sci Nutr.* (2017) 68:167–78. 10.1080/09637486.2016.1226272 27608859

[23] Bártová V Bárta J Brabcová A Zdráhal Z Horáčková V. Amino acid composition and nutritional value of four cultivated South American potato species. *J Food Compos Anal.* (2015) 40:78–85. 10.1016/j.jfca.2014.12.006

[24] Cui R Zhu F. Changes in structure and phenolic profiles during processing of steamed bread enriched with purple sweetpotato flour. *Food Chem.* (2022) 369:130578. 10.1016/j.foodchem.2021.130578 34479007

[25] Fen X Honghai H Xiaofeng D Qiannan L Yanjie H Hong Z. Nutritional compositions of various potato noodles: comparative analysis. *Int J Agr Biol Eng.* (2017) 10:218. 10.3965/j.ijabe.20171001.2287

[26] Gao S Hong J Liu C Zheng X Li L Tian X. Comparative study of different fermentation and cooking methods on dough rheology and the quality of Chinese steamed/baked bread. *J Food Process Pres.* (2022) 46:e16221. 10.1111/jfpp.16221

[27] Kang XM Yu B Zhang HY Sui J Guo L Abd El-Aty AM The formation and *in vitro* enzymatic digestibility of starch-lipid complexes in steamed bread free from and supplemented with different fatty acids: effect on textural and retrogradation properties during storage. *Int J Biol Macromol.* (2021) 166:1210–9. 10.1016/j.ijbiomac.2020.11.003 33157138

[28] Coțovanu I Batariuc A Mironeasa S. Characterization of quinoa seeds milling fractions and their effect on the rheological properties of wheat flour dough. *Appl Sci.* (2020) 10:7225. 10.3390/app10207225

[29] Wang C Yang Z Guo X Zhu K. Effects of insoluble dietary fiber and ferulic acid on the quality of steamed bread and gluten aggregation properties. *Food Chem.* (2021) 364:130444. 10.1016/j.foodchem.2021.130444 34186483

[30] Foste M Jekle M Becker T. Structure stabilization in starch-quinoa bran doughs: the role of water availability and gelatinization. *Carbohyd Polym.* (2017) 174:1018–25. 10.1016/j.carbpol.2017.06.068 28821022

[31] Bressiani J Santetti GS Oro T Esteres V Biduski B Miranda MZD Hydration properties and arabinoxylans content of whole wheat flour intended for cookie production as affected by particle size and Brazilian cultivars. *Lwt.* (2021) 150:111918. 10.1016/j.lwt.2021.111918

[32] Sheikholeslami Z Mahfouzi M Karimi M Ghiafehdavoodi M. Modification of dough characteristics and baking quality based on whole wheat flour by enzymes and emulsifiers supplementation. *Lwt.* (2021) 139:110794. 10.1016/j.lwt.2020.110794

[33] Tuersuntuoheti T Wang Z Zhang M Pan F Liang S Sohail A Different preparation methods affect the phenolic profiles and antioxidant properties of Qingke barley foods. *Cereal Chem.* (2021) 98:729–39. 10.1002/cche.10416

[34] Di Lorenzo C Colombo F Biella S Stockley C Restani P. Polyphenols and human health: the role of bioavailability. *Nutrients.* (2021) 13:273. 10.3390/nu13010273 33477894 PMC7833401

[35] de Araujo FF Farias DDP Neri-Numa IA Pastore GM. Polyphenols and their applications: an approach in food chemistry and innovation potential. *Food Chem.* (2021) 338:127535. 10.1016/j.foodchem.2020.127535 32798817

[36] Rahman MM Rahaman MS Islam MR Rahman F Mithi FM Alqahtani T Role of phenolic compounds in human disease: current knowledge and future prospects. *Molecules.* (2022) 27:233. 10.3390/molecules27010233 35011465 PMC8746501

[37] Chai Y Wang M Zhang G. Interaction between amylose and tea polyphenols modulates the postprandial glycemic response to high-amylose maize starch. *J Agicr Food Chem.* (2013) 61:8608–15. 10.1021/jf402821r 23964645

[38] Zeng X Zheng B Li T Chen L. How to synchronously slow down starch digestion and retrogradation: a structural analysis study. *Int J Biol Macromol.* (2022) 212:43–53. 10.1016/j.ijbiomac.2022.05.099 35597377

[39] Comino P Collins H Lahnstein J Gidley MJ. Effects of diverse food processing conditions on the structure and solubility of wheat, barley and rye endosperm dietary fibre. *J Food Eng.* (2016) 169:228–37. 10.1016/j.jfoodeng.2015.08.037

[40] Tanes C Bittinger K Gao Y Friedman ES Nessel L Paladhi UR Role of dietary fiber in the recovery of the human gut microbiome and its metabolome. *Cell Host Microbe.* (2021) 29:394. 10.1016/j.chom.2020.12.012 33440171 PMC8022197

[41] Cronin P Joyce SA O’Toole PW O’Connor EM. Dietary fibre modulates the gut microbiota. *Nutrients.* (2021) 13:1655. 10.3390/nu13051655 34068353 PMC8153313

[42] Cong J Zhou P Zhang R. Intestinal microbiota-derived short chain fatty acids in host health and disease. *Nutrients.* (2022) 14:1977. 10.3390/nu14091977 35565943 PMC9105144

[43] Spencer CN McQuade JL Gopalakrishnan V McCulloch JA Vetizou M Cogdill AP Dietary fiber and probiotics influence the gut microbiome and melanoma immunotherapy response. *Science.* (2021) 374:1632. 10.1126/science.aaz7015 34941392 PMC8970537

[44] Sun LJ Warren FJ Gidley MJ. Natural products for glycaemic control: polyphenols as inhibitors of alpha-amylase. *Trends Food Sci Tech.* (2019) 91:262–73. 10.1016/j.tifs.2019.07.009

[45] Li YL Lv J Wang L Zhu YY Shen RL. Effects of millet bran dietary fiber and millet flour on dough development, steamed bread quality, and digestion *In Vitro*. *Appl Sci Basel.* (2020) 10:912. 10.3390/app10030912

[46] Zhang S Chen S Geng S Liu CZ Ma HJ Liu BG. Effects of tartary buckwheat bran flour on dough properties and quality of steamed bread. *Foods.* (2021) 10:2052. 10.3390/foods10092052 34574162 PMC8467894

[47] Guo XN Yang S Zhu KX. Influences of alkali on the quality and protein polymerization of buckwheat Chinese steamed bread. *Food Chem.* (2019) 283:52–8. 10.1016/j.foodchem.2019.01.039 30722907

[48] Kou XR Luo DL Zhang KY Xu W Li X Xu BC Textural and staling characteristics of steamed bread prepared from soft flour added with inulin. *Food Chem.* (2019) 301:125272. 10.1016/j.foodchem.2019.125272 31377629

[49] Zhang PP He ZH Chen DS Zhang Y Larroque OR Xia XC. Contribution of common wheat protein fractions to dough properties and quality of northern-style Chinese steamed bread. *J Cereal Sci.* (2007) 46:1–10. 10.1016/j.jcs.2006.10.007

[50] Thakur S Singh N Kaur A. Characteristics of normal and waxy corn: physicochemical, protein secondary structure, dough rheology and chapatti making properties. *J Food Sci Tech.* (2017) 54:3285–96. 10.1007/s13197-017-2775-5 28974814 PMC5602993

[51] Guo L Xu D Fang F Jin Z Xu X. Effect of glutathione on wheat dough properties and bread quality. *J Cereal Sci.* (2020) 96:103116. 10.1016/j.jcs.2020.103116

[52] Li ZJ Deng C Li HF Liu CH Bian K. Characteristics of remixed fermentation dough and its influence on the quality of steamed bread. *Food Chem.* (2015) 179:257–62. 10.1016/j.foodchem.2015.02.009 25722163

[53] Pan L Luo S Liu F Luo J. Comparison of rheological properties of dough and antistaling characteristics of Chinese Steamed Bread containing -glucan from yeast or oat. *Cereal Chem.* (2018) 95:149–57. 10.1002/cche.10020

[54] Liu X Mu T Sun H Zhang M Chen J. Influence of potato flour on dough rheological properties and quality of steamed bread. *J Integr Agr.* (2016) 15:2666–76. 10.1016/S2095-3119(16)61388-6

[55] Tandazo AS Ozturk OK Hamaker BR Campanella OH. Rice starch and Co-proteins improve the rheological properties of zein dough. *J Cereal Sci.* (2021) 102:103334. 10.1016/j.jcs.2021.103334

[56] Czubaszek A Wojciechowicz-Budzisz A Spychaj R Kawa-Rygielska J. Baking properties of flour and nutritional value of rye bread with brewer’s spent grain. *Lwt.* (2021) 150:111955. 10.1016/j.lwt.2021.111955

[57] Zhu F Wang Y. Rheological and thermal properties of rice starch and rutin mixtures. *Food Res Int.* (2012) 49:757–62. 10.1016/j.foodres.2012.09.031

[58] Zhu F. Interactions between starch and phenolic compound. *Trends Food Sci Tech.* (2015) 43:129–43. 10.1016/j.tifs.2015.02.003

[59] Pradeep PM Sreerama YN. Phenolic antioxidants of foxtail and little millet cultivars and their inhibitory effects on α-amylase and α-glucosidase activities. *Food Chem.* (2018) 247:46–55. 10.1016/j.foodchem.2017.11.103 29277227

[60] Ozdal T Capanoglu E Altay F. A review on protein-phenolic interactions and associated changes. *Food Res Int.* (2013) 51:954–70. 10.1016/j.foodres.2013.02.009

[61] Ji X Zeng C Yang D Mu S Shi Y Huang Y Addition of 1, 4-α-glucan branching enzyme during the preparation of raw dough reduces the retrogradation and increases the slowly digestible fraction of starch in cooked noodles. *J Cereal Sci.* (2022) 104:103431. 10.1016/j.jcs.2022.103431

[62] Ben Halima N Borchani M Fendri I Khemakhem B Gosset D Baril P Optimised amylases extraction from oat seeds and its impact on bread properties. *Int J Biol Macromol.* (2015) 72:1213–21. 10.1016/j.ijbiomac.2014.10.018 25453287

[63] Liu W Brennan MA Serventi L Brennan CS. Effect of cellulase, xylanase and α-amylase combinations on the rheological properties of Chinese steamed bread dough enriched in wheat bran. *Food Chem.* (2017) 234:93–102. 10.1016/j.foodchem.2017.04.160 28551272

[64] Fu Z Che L Li D Wang L Adhikari B. Effect of partially gelatinized corn starch on the rheological properties of wheat dough. *Lwt Food Sci Technol.* (2016) 66:324–31. 10.1016/j.lwt.2015.10.052

[65] Li QQ Li C Li EP Gilbert RG Xu B. A molecular explanation of wheat starch physicochemical properties related to noodle eating quality. *Food Hydrocolloid.* (2020) 108:106035. 10.1016/j.foodhyd.2020.106035

[66] Li C Hu YM. Antagonistic effects of amylopectin and amylose molecules on the starch inter- and intramolecular interactions during retrogradation. *Lwt Food Sci Technol.* (2021) 148:111942. 10.1016/j.lwt.2021.111942

[67] Goesaert H Slade L Levine H Delcour JA. Amylases and bread firming – an integrated view. *J Cereal Sci.* (2009) 50:345–52. 10.1016/j.jcs.2009.04.010

[68] Londono DM Smulders MJM Visser RGF Gilissen LJWJ Hamer RJ. Effect of kilning and milling on the dough-making properties of oat flour. *Lwt Food Sci Technol.* (2015) 63:960–5. 10.1016/j.lwt.2015.04.033

[69] Hüttner EK Bello FD Arendt EK. Rheological properties and bread making performance of commercial wholegrain oat flours. *J Cereal Sci.* (2010) 52:65–71. 10.1016/j.jcs.2010.03.004

[70] Buksa K Krystyjan M. Arabinoxylan–starch–protein interactions in specially modified rye dough during a simulated baking process. *Food Chem.* (2019) 287:176–85. 10.1016/j.foodchem.2019.02.077 30857687

[71] Lin L Yang H Chi CD Ma XB. Effect of protein types on structure and digestibility of starch-protein-lipids complexes. *Lwt Food Sci Technol.* (2020) 134:110175. 10.1016/j.lwt.2020.110175

[72] Zou W Sissons M Gidley MJ Gilbert RG Warren FJ. Combined techniques for characterising pasta structure reveals how the gluten network slows enzymic digestion rate. *Food Chem.* (2015) 188:559–68. 10.1016/j.foodchem.2015.05.032 26041231

[73] Zou W Schulz BL Tan XL Sissons M Warren FJ Gidley MJ The role of thermostable proteinaceous alpha-amylase inhibitors in slowing starch digestion in pasta. *Food Hydrocolloid.* (2019) 90:241–7. 10.1016/j.foodhyd.2018.12.023

[74] Melis S Pauly A Delcour JA. Impact of lipases with different substrate specificity in wheat flour separation on the properties of the resultant gluten. *J Cereal Sci.* (2017) 77:291–6. 10.1016/j.jcs.2017.08.024

[75] Miś A Grundas S Dziki D Laskowski J. Use of farinograph measurements for predicting extensograph traits of bread dough enriched with carob fibre and oat wholemeal. *J Food Eng.* (2012) 108:1–12. 10.1016/j.jfoodeng.2011.08.007

[76] Panghal A Khatkar BS Yadav DN Chhikara N. Effect of finger millet on nutritional, rheological, and pasting profile of whole wheat flat bread (chapatti). *Cereal Chem.* (2019) 96:86–94. 10.1002/cche.10111

[77] Ge J Chen X Zhang X Dai Q Wei H. Comparisons of rice taste and starch physicochemical properties in superior and inferior grains of rice with different taste value. *Food Res Int.* (2023) 169:112886. 10.1016/j.foodres.2023.112886 37254334

[78] Leys S De Bondt Y Bosmans G Courtin CM. Assessing the impact of xylanase activity on the water distribution in wheat dough: a 1H NMR study. *Food Chem.* (2020) 325:126828. 10.1016/j.foodchem.2020.126828 32413686

[79] Liu Y Wang X Ren T Ma Z Liu L Li X Effect of oatmeal on texture, water mobility, and starch retrogradation properties of Chinese steamed bread. *Cereal Chem.* (2019) 96:349–57. 10.1002/cche.10133

[80] Sharma B Gujral HS Solah V. Effect of incorporating finger millet in wheat flour on mixolab behavior, chapatti quality and starch digestibility. *Food Chem.* (2017) 231:156–64. 10.1016/j.foodchem.2017.03.118 28449992

[81] Zhou M Li Y Yuan Z Luo L Ren Y Zeng Q Effect of tartary buckwheat and highland barley flours on the quality of glutinous rice cakes determined by nutritional composition, physical structure, sensory perception and flavour. *Int J Food Sci Tech.* (2023) 58:2400–10. 10.1111/ijfs.16377

[82] Wu D Yu L Guo L Li S Yao X Yao Y Effect of highland barley on rheological properties, textural properties and starch digestibility of Chinese steamed bread. *Foods.* (2022) 11:1091. 10.3390/foods11081091 35454677 PMC9025642

[83] Haini N Jau-Shya L Rosli R Mamat H. Effects of high-amylose maize starch on the glycemic index of Chinese Steamed Buns (CSB). *Heliyon.* (2022) 8:e09375. 10.1016/j.heliyon.2022.e09375 35574202 PMC9096677

[84] Lapčíková B Burešová I Lapčík L Dabash V Valenta T. Impact of particle size on wheat dough and bread characteristics. *Food Chem.* (2019) 297:124938. 10.1016/j.foodchem.2019.06.005 31253272

[85] Shumoy H Van Bockstaele F Devecioglu D Raes K. Effect of sourdough addition and storage time on *in vitro* starch digestibility and estimated glycemic index of tef bread. *Food Chem.* (2018) 264:34–40. 10.1016/j.foodchem.2018.05.019 29853385

[86] Li F Guan X Li C. Effects of degree of milling on the starch digestibility of cooked rice during (*in vitro*) small intestine digestion. *Int J Biol Macromol.* (2021) 188:774–82. 10.1016/j.ijbiomac.2021.08.079 34403679

[87] Li H Kerr ED Schulz BL Gidley MJ Dhital S. Pasting properties of high-amylose wheat in conventional and high-temperature rapid visco analyzer: molecular contribution of starch and gluten proteins. *Food Hydrocolloid.* (2022) 131:107840. 10.1016/j.foodhyd.2022.107840

[88] Ma M Mu T Sun H Zhou L. Evaluation of texture, retrogradation enthalpy, water mobility, and anti-staling effects of enzymes and hydrocolloids in potato steamed bread. *Food Chem.* (2022) 368:130686. 10.1016/j.foodchem.2021.130686 34399176

[89] Guo X Dai T Chen M Deng L Chen J Liu C. Steam bread made by superfine purple corn flour: texture characteristics and in vitro starch digestibility. *Lwt Food Sci Technol.* (2022) 169:113967. 10.1016/j.lwt.2022.113967

[90] Wang S Khamchanxana P Zhu F Zhu C Pan J. Textural and sensory attributes of steamed bread fortified with high-amylose maize starch. *J Texture Stud.* (2017) 48:3–8. 10.1111/jtxs.12208

[91] Nkhabutlane P du Rand GE de Kock HL. Quality characterization of wheat, maize and sorghum steamed breads from Lesotho. *J Sci Food Agr.* (2014) 94:2104–17. 10.1002/jsfa.6531 24338919

[92] Aponte M Boscaino F Sorrentino A Coppola R Masi P Romano A. Effects of fermentation and rye flour on microstructure and volatile compounds of chestnut flour based sourdoughs. *Lwt Food Sci Technol.* (2014) 58:387–95. 10.1016/j.lwt.2014.03.022

[93] Mulet-Cabero AI Egger L Portmann R Menard O Marze S Minekus M A standardised semi-dynamic in vitro digestion method suitable for food - an international consensus. *Food Funct.* (2020) 11:1702–20. 10.1039/c9fo01293a 32039430

[94] Wang S Opassathavorn A Zhu F. Influence of quinoa flour on quality characteristics of cookie, bread and chinese steamed bread. *J Texture Stud.* (2015) 46:281–92. 10.1111/jtxs.12128

[95] Both J Biduski B Gomez M Bertolin TE Friedrich MT Gutkoski LC. Micronized whole wheat flour and xylanase application: dough properties and bread quality. *J Food Sci Tech.* (2021) 58:3902–12. 10.1007/s13197-020-04851-2 34471314 PMC8357864

[96] Ghoshal G Shivhare US Banerjee UC. Rheological properties and microstructure of xylanase containing whole wheat bread dough. *J Food Sci Tech.* (2017) 54:1928–37. 10.1007/s13197-017-2627-3 28720949 PMC5495718

[97] Ren S Ma R. Effects of xylanase on quality of frozen dough steamed bread. *Food Sci Technol Res.* (2016) 22:409–17. 10.3136/fstr.22.409

[98] Colakoglu AS Ozkaya H. Potential use of exogenous lipases for DATEM replacement to modify the rheological and thermal properties of wheat flour dough. *J Cereal Sci.* (2012) 55:397–404. 10.1016/j.jcs.2012.02.001

[99] Liu H Zeng F Wang Q Ou S Tan L Gu F. The effect of cryogenic grinding and hammer milling on the flavour quality of ground pepper (*Piper nigrum* L.). *Food Chem.* (2013) 141:3402–8. 10.1016/j.foodchem.2013.06.052 23993499

[100] Saxena SN Sharma YK Rathore SS Singh KK Barnwal P Saxena R Effect of cryogenic grinding on volatile oil, oleoresin content and anti-oxidant properties of coriander (*Coriandrum sativum* L.) genotypes. *J Food Sci Tech.* (2015) 52:568–73. 10.1007/s13197-013-1004-0

[101] Muttakin S Kim MS Lee D. Tailoring physicochemical and sensorial properties of defatted soybean flour using jet-milling technology. *Food Chem.* (2015) 187:106–11. 25977004 10.1016/j.foodchem.2015.04.104

[102] Hussain S Li J Jin W Yan S Wang Q. Effect of micronisation on dietary fibre content and hydration properties of lotus node powder fractions. *Int J Food Sci Tech.* (2018) 53:590–8. 10.1111/ijfs.13632

[103] Zhao Z Mu T Sun H. Comparative study of the nutritional quality of potato steamed bread fermented by different sourdoughs. *J Food Process Pres.* (2019) 43:e14080. 10.1111/jfpp.14080

